# Novichok agents: a historical, current, and toxicological perspective

**DOI:** 10.1080/24734306.2018.1475151

**Published:** 2018-06-29

**Authors:** Peter R. Chai, Bryan D. Hayes, Timothy B. Erickson, Edward W. Boyer

**Affiliations:** a Division of Medical Toxicology, Department of Emergency Medicine, Brigham and Women’s Hospital, Harvard Medical School, Boston, MA, U.S.A; b Department of Emergency Medicine, Massachusetts General Hospital, Harvard Medical School, Boston, MA, U.S.A; c The Fenway Institute, Boston, MA, U.S.A; d Harvard Humanitarian Initiative, Harvard University, Cambridge, MA, U.S.A

**Keywords:** Novichok, nerve agents, organophosphates, chemical weapons

## Abstract

The *Novichok*, or “newcomer” class of nerve agents are lesser characterized, weaponized organophosphate agents. The use of known *Novichok* agents in warfare is banned under the Chemical Weapons Convention of 1997. *Novichok* agents are considered more potent than VX gas and can be applied in unitary and binary forms. Like other nerve agents, *Novichok* agents irreversibly bind acetylcholinesterase and produce a cholinergic toxidrome. Uniquely, these agents are thought to also target neurons in the peripheral nervous system. Delayed treatment or massive exposure may therefore cause a debilitating neuropathy. The recent 2018 assassination attempt of Russian dissident Sergei Skripal and his daughter Yulia in the United Kingdom highlights the importance of recognizing the potential lethal effects of these nerve agents. Treatment of *Novichok* agent poisoning is similar to management of other nerve agents. Given increasing worldwide incidents attributed to chemical weapons such as *Novichok* agents, clinicians should know how to rapidly recognize symptoms of acute poisoning and administer life-saving antidotal therapy, when indicated.

“Circles appeared before my eyes: red and orange. A ringing in my ears, I caught my breath. And a sense of fear: like something was about to happen. I sat down on a chair and told the guys: it’s got me”

––Andrei Zheleznyakov, Russian military researcher after he was exposed to Novichok-5 from a malfunctioning fume hood (1987) [1].

On 4 March 2018, Sergei Skripal, a former Russian military intelligence officer, and his daughter, Yulia Skripal collapsed on a park bench in Salisbury, United Kingdom after eating dinner at a local restaurant [2]. The Skripals were rapidly transported to the hospital where they received treatment for a presumed nerve agent exposure. A responding police officer also developed symptoms of nerve agent poisoning and was admitted to the hospital. A few days later, the media reported that biological sampling of the Skripals confirmed the presence *of Novichok*, a highly potent nerve agent developed as part of the Russian classified nerve agent program, FOLIANT [3]. One month later, scientists from the Organization for the Prohibition of Chemical Weapons (OPCW) confirmed the presence of Novichok in biological sampling from the Skripals as well as from the site of suspected exposure. At the time of this writing, Yulia has fully recovered and was discharged from the hospital, and Sergei remains hospitalized but is expected to recover.

The attempted assassination of the Skripals mirrors the recent assassination of Kim Jong-Nam, brother of North Korean dictator Kim Jong-Un, using ethyl *N*-2-diisopropylaminoethyl methylphosphonothiolate (VX) [4]. The use of *Novichok*, a rare and classified organophosphate, is especially concerning. Here, we review the known toxicity and effects of the *Novichok* class of organophosphate agents and describe the role of toxicologists in advocating for the ban of these chemical weapons as well as global compliance with the 1997 Chemical Weapons Convention that prohibits the large-scale use, development, production, stockpiling, and transfer of chemical weapons and their precursors [5].

Most of what we understand of *Novichok* agents comes from testimony and memoirs of Dr. Vil S. Mirzayanov, the Chief of the Department of Counteraction against Foreign Technical Intelligence at the Russian State Union Scientific Research Institute for Organic Chemistry and Technology (GosNIIOKhT) [6]. Dr. Mirzayanov authored a 1994 report with the Stimson Center describing the state of chemical weapon disarmament in Russia [7]. He detailed the initiation of a secret Soviet chemical weapons initiative to develop “newcomer” (i.e. *Novichok* in Russian) agents. The first three of these, Substance-33, A-230, and A-232, were produced at a GosNIIOKhT facility Russia using an organophosphate structural backbone (Figure 1) [7]. These three newcomer agents were synthesized much like VX, tabun, soman, and sarin, as unitary agents, meaning that the chemical structure is altered during production so that maximum potency occurs rapidly at the outset. Importantly, only binary agents (two inert substances that are combined prior to delivery to create the active nerve agent) that were effectively weaponized and tested were given the designator *Novichok*, while unitary agents and other organophosphates maintain their original designators. For the purposes of this review, we will refer to the newcomer agents by their original designator names. At the same time, the United States recognized the danger of stockpiling potent unitary agents, and initiated development of a Binary Internally Generated chemical weapon in the EYE series of canister weapons (BigEYE) [8]. In response, development of binary newcomer agents escalated at GosNIIOKhT, and in 1989 the first known binary newcomer agent, *Novichok*-5 was synthesized off the base structure of A-232 [7].

*Novichok* class compounds are postulated to be organophosphates containing a dihaloformaldoxime moiety [9]. Several chemical structures purported to be lower potency *Novichok* agents have been published in the scientific literature with the intention of masking the *Novichok* program as a pesticide research program [10–15]. According to Dr. Mirzavanov, and GosNIIOKhT chemist Vladimir Uglev, hundreds of *Novichok* agents were synthesized although only Substance-33, A-230, A-232, A-234, *Novichok*-5, and *Novichok*-7 are known to be weaponized (Table 1) [6,7,16]. Most weaponized *Novichok* agents are believed to be binary. In the case of A-234, the binary agents were reported to be acetonitrile and a low potency organophosphate [17]. *Novichok* agents are also available in liquid form, though they can also be converted into a “dusty” formulation by adsorbing liquid droplets onto a solid carrier like talc, silica gel, fuller’s earth or pumice [18]. Some of these compounds were successfully independently synthesized by the Organization for the Prohibition of Chemical Weapons Central Analytical Database [19].

Binary *Novichok* agents possess several military advantages. First, an individual agent, depending on its chemical subgroups, may not violate the Chemical Weapons Treaty and, hence, is “legal.” These subgroups can be innocuous compounds whose sinister intent may be difficult to detect. Second, the components of binary agents are extremely stable and have limited degradation over time. Third, *Novichok* agents are highly potent, reportedly 5–10 times more so than VX [6,18].

Exposure to *Novichok* agents is fatal unless aggressively managed. The LD50 of *Novichok* agents is reportedly approximately 0.22 mcg/kg similar to 2-(dimethylamino)ethyl *N*,*N*-dimethylphosphoramidofluoridate (VG), a novel, fourth-generation nerve agent [20]. Like other organophosphate compounds, *Novichok* agents bind acetylcholinesterase preventing degradation of acetylcholine and producing the cholinergic or muscarinic toxidrome. The *Novichok*-acetylcholinesterase bond undergoes a similar aging process like other organophosphates, rendering acetyl-cholinesterase inactive and unable to metabolize acetylcholine, resulting in prolonged neurotoxicity and respiratory paralysis. Andrei Zheleznyakov’s description of aerosolized exposure to *Novichok*-5 due to a malfunctioning fume hood in 1987 highlights early toxicity consisting of mydriasis and shortness of breath due to bronchorrhea [1]. These symptoms rapidly progress to seizures, respiratory paralysis, bradycardia, coma, and death. In addition to their cholinergic effects, *Novichok* agents’ binding to peripheral sensory nerves distinguishes this chemical class from organophosphates [7,9]. Prolonged or high-dose exposures are result in a debilitating peripheral neuropathy.

Treatment of *Novichok* or other nerve agent exposure is threefold. First, like any exposure to nerve agents, directed decontamination is important to prevent continued exposure to the patient and to emergency response staff. Clothing exposed to nerve agents can emit trapped vapors for up to 30 min [18]. Although most nerve agents decompose slowly in water, raising the pH of decontamination solution may speed hydrolysis. Use of dry bleach powder should be avoided as it may hydrolyze nerve agents into toxic metabolites [18]. In the case of *Novichok* agents, hydrolysis produces hydrofluoric acid, hydrochloric acid, hydrogen cyanide, and downstream oximes that can continue to produce cholinergic effects to the exposed individuals [18]. Second, like other organophosphate nerve agents, clinicians should immediately administer intravenous (IV) atropine (2–6 mg every 5–10 min) until bradycardia, bronchorrhea and bronchospasm (e.g. muscarinic symptoms) resolve. Induced tachycardia is not a contraindication for escalating atropine doses. Seizures from nerve agent poisoning can be prevented and treated with diazepam. Hospital atropine stocks may be rapidly depleted in the treatment of poisoned victims due to the large dose of atropine typically required to reverse cholinergic symptoms and the volume of patients seeking medical care after a nerve agent attack. Clinicians should recognize possible alternative sources for atropine, including veterinary clinics. When determining which products to stock in a hospital’s emergency drug cache, consideration should be given to higher concentration atropine (1 mg/mL) for mass casualty situations involving potent nerve agent exposure. Possible adjunctive therapies, although only tested in animal models, include diphenhydramine, and in critically ill individuals, IV lipid emulsion [4,21,22]. Third, antidotal therapy in the form of IV administration of 1–2 g of pralidoxime (2-PAM), or 250 mg of obidoxime is indicated to restore acetylcholinesterase activity [23]. To maintain adequate plasma concentrations, pralidoxime should be administered every 3–6 h or as a continuous infusion for at least 24 h after the last dose of atropine [24]. Antidotes (e.g. 2-PAM or other oximes) must be administered before the aging process occurs. Because the toxicity of *Novichok* agents appears not to primarily rely on acetylcholinesterase inhibition, some have suggested that reactive oximes like potassium 2,3-butanedione monoximate (available in Europe only) are the preferred oximes for antidotal therapy [25,26].

The fact that Sergei and Yulia Skripal survived poisoning from a *Novichok* agent is a testament to the astute diagnostic skills of the responding clinicians. This case underscores the importance of recognizing the cholinergic toxidrome, understanding its etiologies and causative agents, instituting proper decontamination measures, and promptly administering antidotal therapy. As toxicologists, we are well-positioned to educate our colleagues in the recognition and treatment of nerve agents. Additionally, our role as advocates for our patients must continue–in a world where chemical weapon assassinations and brazen nerve agent attacks against innocent civilians appear to be becoming frequent, we must use our voice to condemn the use of these and other banned chemical weapons [5]. Furthermore, we should also be cognizant of the confounding manipulation of scientific literature and lay media to distort the lens in which we view chemical weapons. Like the publication of peer-reviewed manuscripts potentially used to mask research and development of *Novichok* agents in the 1990s, today’s modern media can be used to distort the facts of chemical weapon incidents. In a world where news of assassinations and chemical attacks is rapidly disseminated, we will increasingly be called upon to lend our expertise in the education, diagnosis, and treatment of nerve agents. We urge our fellow toxicologists and clinicians to continue to speak out, defend, and develop policies against the use of chemical weapons.

## Figures and Tables

**Figure 1:**
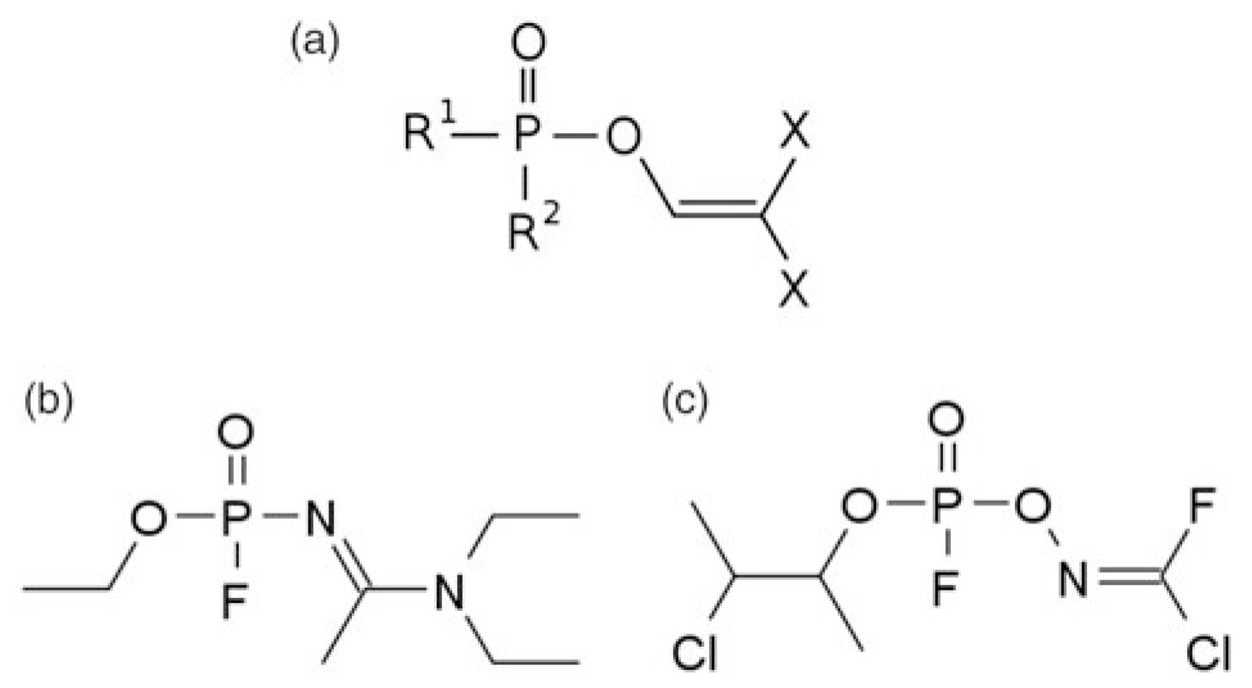
(a) Organophosphate structural backbone of *Novichok* agents. R =alkyl, alkoxy, aklylamino, or fluorine; X =halogen (F, Cl, Br) or pseudohalogen (CN). (b) Chemical structure of A-234 as described by Mirzayanov, and (c) by Hoenig and Ellison [18,27].

**Table 1:** A list of known Novichok agents attributed to the GosNIIOKhT research program and their status.

Agent	Type	Current status
substance-33	Unitary	Estimated 15,000 tons produced. Designated as chemical weapon
A-230	Unitary	Experimental quantities produced. Designated chemical weapon (1990)
A-232	Unitary	Experimental agent. Not designated, or officially approved
A-234	Unitary analog of A-232	Unknown
Novichok-5	Binary analog of A-232, 8× more effective than VX	Experimental agent. Designated chemical weapon (1989)
Novichok-7	Binary analog of A-234, 10× more effective than soman	Experimental agent. Not designated
Novichok-# (no established designator)	Binary analog of Substance-33	Adopted as chemical weapon (1990)
